# Reasons for Fearing the Appearance of Cholera or Other Epidemic

**Published:** 1865-10

**Authors:** 


### Editorial Department.

### Reasons for fearing the appearance of Cholera or other Epidemic.

There appears to be a prevailing fear, both with the profession and public, that cholera is again to visit our country, growing out of the excitement regarding its prevalence in various places on the continent of Europe, its rumored appearance in England, and the history of its former rise and progress from the old world westward to the new. This is perhaps sufficient ground for fears that we shall not long remain exempt from the disease; but there are other reasons much more potent, which should make us fear, in our large cities, the outbreak of cholera, or some equally alarming and fatal epidemic. All observation and experience go to prove that epidemic disease is induced mainly by local causes, and that the absence of proper sanitary regulations in our large cities constitutes a much greater ground of apprehension than the history and progress of any former outbreak of disease. The sanitary condition of New York is reported as fearful beyond all parallel; the filth and stench of the lower and crowded portions of the city being indescribably disgusting. In this great cess-pool of corruption, may at any time be generated the poisons, of epidemic disease, cholera, plague, fever, any of the forms of disease which such causes generate are liable to spring up, named, to be sure, by accidental circumstances, but still always the same, producing death in fearful numbers, and from almost uniform causes. From New

York and other large commercial centers, epidemic diseases radiate; wherever similar food is prepared, thither would spread the fatal plague.

New York, Philadelphia, Boston and New Orleans, are not alone centers for the spread of epidemic disease, and we had not proposed to speak of other cities, but more especially to suggest some causes for apprehension in our own, as it is called, cleanly and beautiful city. Buffalo is situated, hygienically, in unsurpassed beauty and attraction, and ought to be the healthiest city in the world; our streets are broad, well paved and drained, and fanned by a lake breeze, not so bleak as represented, still strong and healthy; they are washed and swept, and perhaps no city in the world can boast of finer, cleaner streets, or more perfect ventillation. As in all cities there is a fine outside show upon the principal streets, but upon the inside, underside and backside there is an untold amount of corruption. It is not in the better portion of the city we look for the generation of disease, but even here we shall be astonished at the frequency of sanitary neglect. It is not from London or New York or other city, that the people of Buffalo may expect cholera or plague, or other fatal epidemic disease; it is not coming from these sources, half so certainly as from their own alleys and yards and vaults (?) (if pyramids are vaults, then we may call these stacks in the outhouses vaults.) Cholera and typhoid fever are no doubt coming—are indeed already here in incipient stage, and unless our board of health look after and remove these causes of disease, and do it now, while the season is favorable for such regeneration, it should be ltself removed, and the citizens commence immediately, the labor properly belonging to the board of health. We understand that the sewerage is also very imperfect, not from lack of what are called sewers, but from complete obstruction, by years of accumulation. We know that many of the lesser branches are completely obstructed, and we hear from the most reliable sources, that the "mains" are also in many instances in the same condition. Worse still, the city is now ordering streets paved and improved and wholly neglecting the important subject of drainage; this is being done in one instance upon a street where is is situated one of our greatest and most important public charities; where the sick are sometimes congregated in great numbers, and where in case of epidemic still greater numbers would almost certainly be conveyed for temporary relief. This institution has suffered for want of sewerage, beyond all estimate; its medical staff and other officers have repeatedly protested against its condition, and several deaths have occurred, almost certainly tracable to sanitary neglect; notwithstandiug all this, we see the street paved and made to present a fine exterior, *without the construction of a sewer*—the very thing above all others which the health of the neighborhood and city required.

If, as a city, we neglect the common decencies of civilization and allow the town to grow foul with unhealthy and sickening odors, the public charities to become pestilential nuisances, and private residences whited walls, partially concealing the unwashed, uncleaned, unsewered yards, from which continually emanate disgusting and deadiy odors, then we may well dread and fear the outbreak of pestilence.

It will appear to some that we are overdrawing our sanitary condition, and that we are not so bad as we represent; on the contrary one-half is not told. Physicians—the guardians of the public health, may speak plainly and in season, but party and office, control everything, "though one rise from the dead." his voice would not be heard; we have official prophets and apostles, we must hear them. We hope to hear them and observe their works. The present time of comparative health should not be allowed to pass without the most thorough washing, draining and ventilating. All the sources of impurity should be removed, such as open-mouthed, obstructed drains, overflowing vaults, open cess-pools, etc., etc., a vast number of which exist, and are a fruitful source of disease at all seasons; in a time of epidemic, a thousand fold increased for evil.

Our citizens manifest some anxiety upon the subject of cholera by frequent inquiries as to the probability of its appearance next season as has been predicted. Upon this point we do not so much desire to remark as upon the other and more practical question; how shall the virulence of epidemic disease be abated? for, whether cholera appear or not, fatal disease will certainly and in vast numbers of instances be the effect of total neglect of sanitary precautions. Other towns and cities have already commenced this work of protection, and we hope that Buffalo will not remain unmoved, when every motive of humanity urges to immediate and energetic action; it is a measure which can now be safely adopted, but the spring and warm season upon us, and our time is past; the stagnant sources of disease are safer, if left unstirred.

